# Gypenoside XLIX inhibiting PI3K/AKT/FOXO1 signaling pathway mediated neuronal mitochondrial autophagy to improve patients with ischemic stroke

**DOI:** 10.3389/fphar.2025.1600435

**Published:** 2025-08-21

**Authors:** Yonglei Liu, Hongdie Mao, Zhengguang Sha, Jishuai Zhao, Hui Cai, Rong Xi, Zhenzhu Zhao, Xiaoling Yin, Lin Yang, Changyun Liu

**Affiliations:** ^1^ Department of Neurology, Fujian Medical University Union Hospital, Fuzhou, Fujian, China; ^2^ Department of Neurology, The First Affiliated Hospital of Dali University, Dali, Yunnan, China; ^3^ Institute of Clinical Neurology, Fujian Medical University, Fuzhou, Fujian, China

**Keywords:** ischemic stroke, gypenoside XLIX, mitochondrial autophagy, FoxO1, PI3K/AKT

## Abstract

**Introduction:**

Ischemic stroke is a leading cause of mortality and disability worldwide, with limited therapeutic options and high rates of recurrence. Mitochondrial dysfunction plays a critical role in neuronal injury during ischemia-reperfusion, making mitochondrial autophagy a potential therapeutic target. Gypenoside XLIX, a major active metabolite of Gynostemma pentaphyllum, exhibits antioxidant and organ-protective properties, but its effects on neuronal mitochondrial damage in stroke remain unclear. This study aimed to explore the neuroprotective mechanisms of Gypenoside XLIX in ischemic stroke, focusing on the PI3K/AKT/FOXO1 signaling pathway.

**Methods:**

Neuroprotective effects were evaluated in oxygen-glucose deprivation (OGD) neuronal cells and middle cerebral artery occlusion (MCAO) rat models. Cell viability, apoptosis, ROS production, mitochondrial membrane potential, and autophagic flux were assessed by CCK-8, flow cytometry, ELISA, TMRE staining, immunofluorescence, and Western blotting. Signaling pathway involvement was examined using PI3K inhibitor LY294002, AKT activator SC79, and FOXO1 knockdown.

**Results:**

Gypenoside XLIX significantly improved neuronal viability (p < 0.01), reduced apoptosis (p < 0.01), and decreased ROS levels (p < 0.001) in OGD cells. It enhanced p-PI3K and p-AKT expression while suppressing FOXO1 (p < 0.05), promoted Beclin-1, LC3, PINK1, and Parkin expression (p < 0.001), and reduced p62 (p < 0 .01). In MCAO rats, Gypenoside XLIX decreased infarct volume (p < 0.001), brain edema (p < 0.01), and TUNEL-positive cells (p < 0.001), while elevating mitochondrial membrane potential and antioxidant enzyme levels (SOD, GSH-Px, CAT; all p < 0.001).

**Conclusion:**

Gypenoside XLIX alleviates ischemic stroke injury by activating the PI3K/AKT/FOXO1 pathway, enhancing mitochondrial autophagy, and reducing oxidative stress, supporting its potential as a novel neuroprotective agent in stroke management.

## 1 Introduction

Stroke is one of the major causes of death and disability around the world. According to from the World Stroke Organization, 13 million people have strokes every year, leading to approximately 5.5 million deaths (Dritsas and Trigka, 2022). In 2020, research in China assessed the significant burden of stroke, with ischemic stroke being notably prevalent. It transpires when the brain’s blood flow is interrupted, resulting in the death of brain cells due to oxygen and nutrient deprivation(Herpich and Rincon, 2020; Rabinstein, 2020). Present-day clinical treatments for stroke encompass thrombolytic therapy employing tissue plasminogen activator (tPA) for ischemic strokes, endovascular thrombectomy for clot removal, and surgical interventions for hemorrhagic strokes (Putaala, 2020). Nonetheless, these treatments come with limitations, such as restricted therapeutic windows, the potential for complications, and their unsuitability for all patients. Moreover, a significant number of patients continue to suffer from long-term disabilities and face high recurrence rates (Tu et al., 2023). These limitations highlight the need to explore new mechanisms of stroke and develop novel drugs that can target these underlying issues more effectively, ultimately improving patient outcomes and quality of life.

*Gynostemma pentaphyllum* (Thunb.) Makino [Cucurbitaceae], as a medicinal material with a long history and unique effects, has always attracted much attention (Su et al., 2021). Its active metabolites include saponins, sterols, flavonoids, polysaccharides, amino acids, and various other metabolites (Xie et al., 2024a). Many clinical and basic experiments have confirmed that these substances have anti-aging, anti-fatigue, cardio-protective, lipid-lowering, liver-protecting, sugar metabolism-improving, and anti-ulcer effects (Li et al., 2023). Among them, Gynostemma pentaphyllum saponins were considered to effectively clear free radicals in the body through their strong antioxidant effects, preventing damage to biomacromolecules such as cell membranes and DNA, thus delaying aging and preventing diseases. Gypenoside XLIX is an important metabolite of Gynostemma pentaphyllum saponins (Liu et al., 2021). Previous studies mainly focused on the protective effects of Gypenoside XLIX on the liver and kidney. For example, it alleviates acute liver injury by modulating relevant pathways and acts as a PPAR-α activator to reduce inflammation. Its kidney protection effects, especially in diabetic nephropathy, have also been explored by improving renal function through down-regulating pro-inflammatory mediators and signaling pathways (Hassan et al., 2021; Zhou et al., 2024). Although it has been confirmed that Gypenoside XLIX has a protective effect on mitochondria, whether the same protective effect exists in the mitochondrial damage induced by ischemia/reperfusion in neuronal cells has not yet been elucidated. Whether the protective effect is mediated by a single saponin or a combination, and the underlying mechanisms, remain unclear. Therefore, an in-depth analysis of the precise functions of Gynostemma pentaphyllum saponins in ischemic stroke is of considerable medical value. This research has the potential to unveil endogenous defense mechanisms and to discover new methods for the treatment of ischemic stroke.

The mitochondria not only serve as the energy source by producing ATP to power various biochemical processes but also undertake a variety of crucial cellular functions (Zheng et al., 2019). When mitochondrial function is compromised, it can become a marker of neuronal death during ischemia-reperfusion (Ajoolabady et al., 2021). Therefore, maintaining the normal operation of mitochondria is vital for protecting neuronal life activities. Studies have confirmed that the Human PI3K and AKT complex, known as the PI3K/AKT signaling pathway, is a cellular messaging pathway closely related to phosphatidylinositol molecules and follows the signaling transduction mechanism of receptor tyrosine kinases (Chen et al., 2022). In the context of ischemic stroke, this pathway is critically involved in neuronal resistance to oxidative stress and mitochondrial dysfunction. FOXO1, a downstream target of AKT, promotes neuronal apoptosis by upregulating pro-apoptotic genes (e.g., Bim, PUMA) and can be inhibited via AKT-mediated phosphorylation, thereby reducing cell death (Ye et al., 2023). Furthermore, numerous studies have verified that this pathway participates in the modulation of mitochondrial biogenesis, mitophagy, as well as the regulation of intracellular redox reactions (Chen et al., 2023a).

Mitophagy, as a selective autophagic process, plays a critical role in maintaining mitochondrial quality and cellular homeostasis (Park et al., 2021). When mitochondria were damaged, such as by oxidative stress induced by ischemia-reperfusion, mitophagy is activated (Zhang et al., 2020). The PINK1/Parkin pathway is a classical regulatory pathway for mitophagy: a decline in the mitochondrial membrane potential of damaged mitochondria leads to the accumulation of PINK1 on the outer mitochondrial membrane, which in turn recruits Parkin. Parkin ubiquitinates outer mitochondrial membrane proteins, prompting autophagosomes to recognize and engulf damaged mitochondria, which were eventually degraded through fusion with lysosomes (Li et al., 2022a; Li et al., 2022b). In the context of ischemic stroke, imbalanced mitophagy exacerbates neuronal injury, while the PI3K/AKT signaling pathway participates in mitophagy regulation by modulating downstream molecules such as the FOXO1 transcription factor to influence the expression of mitophagy-related genes (Shen et al., 2018; Wang et al., 2022).

The objective of this research was to explore the specific molecular mechanisms and modalities of Gypenoside XLIX in the treatment of ischemic stroke. We investigated the mechanism of action of Gypenoside XLIX using MCAO animal models and OGD-induced cell injury models. Assays included Western blotting, immunofluorescence, and RT-PCR to analyze protein and gene expression levels. In addition, signaling pathway inhibitors and activators or FOXO1 gene knockdown were used to clarify the function of the PI3K/AKT signaling pathway and FOXO1 in Gypenoside XLIX protection against stroke.

## 2 Materials and methods

### 2.1 Bioinformatics analysis

The GSE22255 dataset was retrieved from the GEO database to analyze the blood genomic expression profile for ischemic stroke (IS) using R language (version 4.3.1, run 3 times independently). To explore potential targets of Gypenoside XLIX (a dammarane-type glycoside, *Gynostemma pentaphyllum* (Thunb.) Makino [Cucurbitaceae] main metabolites; CAS: 94987-08-3; Formula: C_52_H_86_O_21_; 99.99% purity; Catalog number: HY-N1990; MedChemExpress), we queried databases including TCMSP, TCMDatabase@Taiwan, and TCMID for predicted target genes. It is crucial to note that these *in silico* predictions serve as hypothesis-generating tools, and subsequent experiments (OGD cell model and MCAO rats) were conducted to validate the biological relevance of these targets. A Venn diagram of the downregulated genes from dataset GSE22255 and the corresponding targets of Gypenoside XLIX was created using Bioinformatics & Evolutionary Genomics tools on Windows 10 OS. Molecular docking experiments were performed with AutoDock Tool (default parameters), and results were visualized using Pymol.

### 2.2 Cell culture

Rats were placed on a laminar flow hood that had been wiped with 75% ethanol. We used ophthalmic scissors to incise the skin and bone along the midline of the brain. The brain tissue was then taken out and the corresponding part of the brain tissue was dissected. The vascular membrane on the surface of the hippocampus was removed, and the crescent-shaped hippocampus was cut into small pieces. These fragmented tissues were placed in a culture dish containing 3 mL of DMEM-F12 (Gibco, United States, Cat. No. 11330032) and 3 mL of 0.25% trypsin for digestion. After centrifugation, the sedimented cells were collected. The counted cells were then inoculated into the culture medium for observation. Finally, immunofluorescence detection was used to identify whether the primary hippocampal neurons from Sprague-Dawley rats were successfully isolated. The passage number was controlled at ≤5.

### 2.3 OGD cell model

Neuronal cells were cultivated within a complete culture medium that comprised 10% FBS. Subsequently, they were positioned in an incubator with an atmosphere of 5% CO_2_ and 95% air, and then incubated at a temperature of 37°C. After washing with PBS, cells were incubated in sugar-free medium in a hypoxic incubator (O_2_ level monitored via built-in gas analyzer, maintained at 3% ± 0.5%, 5% CO_2_, 92% N_2_) at 37°C for 8 h. Then change to a fresh complete medium and transfer the cells to a normal oxygen content incubator. Incubate at 37°C for 12 h. Simulate *in vivo* ischemic conditions through sugar-free and hypoxic culture, and then transfer to normal medium to simulate *in vivo* reperfusion conditions with normal oxygen content, thereby constructing the OGD model.

### 2.4 Cell transfection

Select cells in the logarithmic growth phase for transfection. Cells were seeded at a density of 5 × 10^5^ cells/well in 6-well plates and transfected when reaching 70%–80% confluence. Si-FOXO1 (targeting FOXO1 mRNA) and si-NC (negative control, non-targeting scrambled sequence) were obtained from GenePharma, located in Shanghai, China. The siRNA sequences were as follows: si-FOXO1-1: 5′-AGC​AAA​UUU​ACU​GUU​GUU​GUC-3’; si-FOXO1-2: 5′-AUC​AUU​UUG​UUA​UGA​GAU​GCC-3’; si-NC (negative control): 5′-CAC​CGT​TCT​CCG​AAC​GTG​TCA​CGT​TTC​AAG​AGA​ACG​TGA​CAC​GTT​CGG​AGA​ATT​TTT​TG-3’. The transfection process was carried out with the Lipofectamine 2000 reagent (manufactured by Invitrogen in California, United States), following the guidelines provided by the manufacturer. Cells were incubated with transfection complexes for 6 h, after which the medium was replaced with fresh complete medium. Transfected cells were harvested 48 h later to detect transfection efficiency by RT-qPCR.

### 2.5 MCAO rats model construction

Sprague Dawley (SD) rats were procured from the National Rodent Seed Center in Shanghai, China. The rats had weights ranging from 200 g (inclusive) to 250 g (exclusive). They were housed in a feeding box maintained at 25°C with a humidity level between 50% and 60%. The light and dark cycle within the feeding box was set at 12 h each. The rats had unrestricted access to feed and water. All the procedures were stringently approved by the animal protection and utilization committee of our institution (No. DFY20230905001).

Rats should be fasting for 12 h before the procedure. Prepare the rat model of left middle cerebral artery occlusion (MCAO) by matching the appropriate suture (4–0 nylon monofilament, Doccol, United States) to the rat’s weight. Intraperitoneal injection anesthesia is performed using 2.5% sodium pentobarbital (dose: 40 mg/kg). Once the anesthesia is successful, place the rat in a supine position and secure it. Shave the hair from the surgical site, disinfect it with iodine, and make a median incision in the neck about 2–3 cm in length. Subsequently, perform a blunt dissection of the muscles and tissues to reveal the common carotid artery (CCA), external carotid artery (ECA), and internal carotid artery (ICA), taking care to avoid pulling on the vagus nerve, and meticulously strip the fascia from the surface of the vessels. Loop the proximal end and bifurcation of the common carotid artery, place a surgical thread in a living knot at the distal end of the CCA, clamp the origin of the internal carotid artery with a hemostat, and pull up the loop at the CCA site. Then, make a small incision about 1 cm from the bifurcation of the CCA towards the heart, insert the prepared suture into the left common carotid artery, and advance it until you encounter resistance upon reaching the origin of the ICA. Ischemia was confirmed by reduced cerebral blood flow monitored with a laser Doppler flowmeter (PeriFlux System 5,000, Perimed, Sweden) below 20% of baseline. Stop advancement at this point. Then, tie the reserved thread at the CCA site to fix it prevent it from coming out, and relax the external carotid artery. After occlusion for 2 h, remove the suture. Then, clean and layer suture the rat’s tissues and skin. Place the rat in a separate cage immediately post-surgery, in a lateral position.

### 2.6 Modified NSS score scale

In this study, researchers who were unaware of the study’s design used a modified NSS scoring method to assess the neurological deficits in the various groups of rats. We conducted the modified NSS neural function scoring according to the content of Supplementary Table S1. The evaluation system includes a total of 3 mutually independent test items, as shown in Supplementary Table S1, which were as follows: (1) motor test. (2) sensory test. (3) loss of reflexes and abnormal movement. Each test item in these projects is scored from 0 to 6, and the scores of each sub-item were added up to get the total score. In this scoring system, the total score ranges from 0 to 18 points. Scores falling within the range of 13–18 points signify severe damage, scores between 7–12 points suggest moderate damage, and scores from 1 - 6 points denote mild damage.

### 2.7 Detection of infarct volume after ischemic stroke

After scoring the neurological function, 10% hydrochloric acid was used to deeply anesthetize the rats, and preoperative preparations were made. The thoracic cavity and heart of the rats were fully exposed, a catheter was left in place, and the heart was punctured from the left ventricle into the ascending aorta. The right auricle was incised, and the rat was rapidly perfused with saline. After the perfusion was completed, the brain was quickly removed, the surface blood was cleared with saline, and then it was placed in a refrigerator (−20°C) for 20 min of freezing. After thawing, the brain hemispheres and other tissues were removed, and the brain was cut into 2 mm sections from the coronal plane. These sections were then placed in a 2% TTC solution and heated in a water bath, with attention paid to ensure proper mixing for thorough staining, for 30 min. Observation of the staining results showed that normal brain tissue turned red, while the ischemic area appeared gray-white. Subsequently, the sections were fixed with 4% paraformaldehyde. After 24 h, images were gathered using Image Proplus 6.0 software to assess the infarct area and the total area of each brain slice. The infarct volume ratio for each rat was calculated as follows: (Total infarct area of brain slice/Total area of brain slice) × 100%.

### 2.8 Hematoxylin-eosin staining

Hematoxylin-eosin staining (Klamar, Shanghai, China) was used to fix the rat brain structure (especially the region around the preoptic commissure from the coronal section). After that, the specimens were embedded in paraffin. Then, cut it into 5 μm sections. Hematoxylin and eosin were employed for staining. Observations were made using a BX51 optical microscope.

### 2.9 Rats brain water content detection

First, a craniotomy is meticulously performed on the rat to extract the brain. Subsequently, the brain is cleansed with saline solution to eliminate blood and surface impurities. Then, filter paper is employed to absorb the moisture on the surface of the brain to minimize its interference with the measurement results. we precisely measure the wet weight of the brain, that is, the weight of the brain when it is in a moist condition. After that, the brain tissue is placed in a temperature-controlled drying oven and dried for 72 h at 105°C to ensure that all the water in the brain is completely evaporated, obtaining the dry weight of the brain, which is the weight in a dry state. Ultimately, the brain water content (expressed as a percentage) is computed using the following formula: (wet weight - dry weight) divided by wet weight, and then the result is multiplied by 100%. This calculation allows for the determination of the proportion of water within the brain tissue.

### 2.10 TUNEL inspection procedure

For the assessment of neuronal apoptosis in the hippocampus within sections, the TUNEL staining method was employed. The cells were initially incubated with a TUNEL mixture. Subsequently, the sections underwent three rounds of washing and were then stained with DAPI. Thereafter, the sections were observed under a fluorescence microscope. Finally, flow cytometry was used to conduct a quantitative analysis of the results.

### 2.11 RT-qPCR

Total RNA was extracted from cells and human serum with TRIzol reagent (manufactured by Invitrogen in Carlsbad, California, United States). The addition of gDNA Eraser (produced by TaKaRa in Liaoning, China) assisted in the elimination of genomic DNA. Reverse transcription of the total RNA was carried out using SuperScript IV (from ThermoFisher). The RT-qPCR analysis was performed on the ProFlex™ PCR system (ThermoFisher) with the One Step SYBR^®^ PrimeScript RT-PCR Kit (TaKaRa). The PCR primers were presented in Supplementary Table S2.

### 2.12 Cellular thermal shift assay (CETSA)

CETSA was performed to validate the direct interaction between Gypenoside XLIX and PI3K. Briefly, rat primary hippocampal neurons were treated with 12.5 μM Gypenoside XLIX or 1% DMSO for 4 h, followed by lysis and aliquoting. The samples were heated at temperatures ranging from 42°C to 69°C (10 min per temperature), centrifuged (20,000 × g, 20 min, 4°C), and subjected to Western blot analysis to determine PI3K protein levels. Band intensities were quantified, and melting curves were generated to assess the shift in melting temperature (ΔTm) between treatment groups.

### 2.13 Western blot analysis

Cells and tissues were lysed using RIPA Buffer Concentrate (produced by Cayman Chemical in the State of Michigan, United States). The total protein was then transferred to SDS - PAGE for constant - pressure electrophoresis lasting 120 min. The isolated proteins were further transferred to the Immobilon - E − PVDF membrane (manufactured by Merck in Darmstadt, Germany). The membrane was incubated with primary antibodies at 4°C for 12 h and with secondary antibodies for 2 h. Enhanced chemiluminescence was employed to visualize the proteins. The antibodies utilized in our study were as follows: FOXO1 (ab39670, 1:500, Abcam), anti-PI3K antibody (ab302958, 1:1,000, Abcam), AKT (ab38449, 1:500, Abcam), p-PI3K (ab151549, 1:1,000, Abcam), p-AKT (ab8805, 1:1,000, Abcam), caspase9 (ab184786, 1:1,000, Abcam), caspase3 (ab184787, 1:2000, Abcam), Drp1 (ab184247, 1:1,000, Abcam) and beta-actin (ab115777, 1:200, Abcam). Goat Anti-Rabbit IgG H&L (HRP) secondary antibodies (ab7090, 1:5,000, Abcam).

### 2.14 ELISA assays

This experiment aims to detect the content of specific biomolecules. The ELISA kits for SOD, CAT, and GSH-PX purchased from Biovision Company in California, United States were selected. The entire experiment is strictly operated. The experimenters take out the specific kit’s balanced reagents in a standardized environment, dilute the standard products and add samples according to the instructions, and incubate the plate. After incubation, operations such as washing, incubating with enzyme conjugate, washing again, adding substrate for color development, adding stop solution, etc., were performed. Finally, the absorbance is measured with an enzyme marker and the content of the target molecule is calculated. The entire process follows the kit instructions to ensure accurate results.

### 2.15 CCK-8

Transfected OGD cell models were introduced into 100 μL of DMEM medium supplemented with FBS. Then, 10 μL of CCK-8 solution (Beyotime) was added. After incubation according to the manufacturer’s guidelines, the absorbance at 450 nm was measured to determine cell viability. This method allows for a quantitative assessment of the metabolic activity of cells.

### 2.16 Flow cytometry apoptosis assay

To determine the apoptotic status, the Annexin V - eGFP/PI Kit (from Genenode, Wuhan, China) was employed. Cells were initially washed with PBS, and 100 µL of cell suspension was transferred to a 5 mL flow tube. Subsequently, 5 µL of Annexin V/eGFP was added to the samples, which were then incubated at room temperature for 5 min in the dark. After adding 10 µL of PI and 400 µL of PBS, the samples were analyzed using the FACSVERSE flow cytometry (from BD Biosciences, CA, United States). Data acquisition and analysis were performed with Flowjo software (also from BD Biosciences). This process enables the accurate determination of the pyroptosis status of cells through the detection of specific markers and the use of advanced flow cytometry techniques and dedicated software for data interpretation.

### 2.17 Detection of ROS levels

Neuronal cells were incubated with 0.25% pancreatic enzyme and gently shaken to digest the cells. The cells were then transferred to EP tubes and centrifuged at 300 *g* in a 4°C centrifuge. After OGD treatment for 4 h, cells were reoxygenated in complete medium with or without Gypenoside XLIX for 24 h. At this time point, each sample was resuspended in serum-free DMEM culture medium containing 10 μmol/L DCFH-DA probe (prepared by diluting DCFH-DA stock solution 1:1,000), resulting in a final concentration of approximately 1 × 10^6^ primary neurons/mL in the flow tube. The mixture is incubated in a 37°C incubator in the dark for 20 min, with mixing every 5 min. Subsequently, the cells undergo three rounds of washing with serum-free DMEM culture medium. This washing process aims to eliminate any remaining fluorescent probe that has not been internalized by the cells, ensuring accurate and reliable results in subsequent analyses. Finally, the fluorescence intensity of each sample was detected using a FACSCantoII flow cytometer (excitation wavelength 488 nm, emission wavelength 525 nm). Data were expressed as the mean fluorescence intensity relative to the control group to analyze the production of ROS in primary neurons of each group.

### 2.18 Detection of intracellular calcium concentration using fluo-3/AM calcium fluorescent probe

When detecting intracellular calcium concentration using the Fluo-3/AM (S1056, Beyotime, China) probe, cells were first seeded into 24-well plates and cultured until they reach 80% confluence. After rinsing with Hepes buffer, Fluo-3/AM with a final concentration of 8 μmol/L (prepared by diluting a 2 mM DMSO stock solution at a ratio of 1:250) is added. The plates were then incubated in the dark for 45 min at 37°C and 5% CO_2_ in a thermostatic shaking incubator at a rotational speed of 50–80 rpm. After incubation, the cells were rinsed three times with Hepes buffer to remove free probes. Images were acquired under a fluorescence microscope using an excitation wavelength of 488 nm and an emission wavelength of 525 nm. Five random fields of view were selected for each sample, and the fluorescence intensity is quantified by integral optical density using ImageJ software and normalized to the control group.

### 2.19 Immunofluorescence staining

For immunofluorescence detection, cells in the logarithmic growth phase were seeded into 6-well plates at a density of 1 × 10^6^ cells per well and treated according to the experimental groups. The supernatant was then discarded, and the cells were gently rinsed 3 times with PBS. Subsequently, 1 mL of 4% paraformaldehyde was added, and the cells were fixed on a shaker (50 rpm) for 20 min. After discarding the supernatant and rinsing 3 times with PBS, 1 mL of 0.25% Triton-X100 was added, and the cells were permeabilized on a shaker (50 rpm) for 20 min. Following another 3 rinses with PBS, 1 mL of goat serum was added for blocking at room temperature for 30 min. The blocking solution was removed, and 1 mL of primary antibodies (Anti-TOMM20/LAMP2, 1:250, ab186735/ab13524, Abcam) were added, followed by overnight incubation at 4°C. After recovering the primary antibodies, the cells were washed 3 times with TBST (5 min each), and 1 mL of secondary antibodies (Goat Anti-Rabbit IgG H&L (Alexa Fluor^®^ 647/488), ab150079/ab150077, Abcam) were added. The cells were incubated on a shaker (50 rpm) in the dark at room temperature for 1 h. The secondary antibodies were discarded, and the cells were washed 3 times with TBST (5 min each). After DAPI staining for 5 min and subsequent removal of DAPI, the cells were washed 3 times with TBST (5 min each). Finally, the coverslips were mounted with Fluoroshield^®^ TM containing DAPI, photographed under a fluorescence microscope, and the fluorescence intensity was analyzed using ImageJ software (NIH).

### 2.20 Mitochondrial membrane potential level

For brain tissue, ischemic brain samples were minced and digested with 0.125% trypsin (1,590–046; Invitrogen) at 37°C for 15 min, followed by termination of digestion with DMEM containing 10% fetal bovine serum. The cell suspension was centrifuged at 1,000 *g* for 5 min, and the pellet was resuspended in PBS to obtain single-cell suspensions. For cell cultures, logarithmic-phase cells (1 × 10^6^ cells/well in 6-well plates) were treated according to experimental groups, washed once with PBS, and incubated with TMRE staining working solution (prepared by diluting TMRE 1000× stock solution to 1× with HBSS buffer) at 37°C for 30 min in the dark. After incubation, brain cell suspensions and cultured cells were centrifuged (1,000 × g, 5 min) or washed twice with preheated PBS/medium, respectively. Fluorescence was visualized using a fluorescence microscope with an excitation wavelength of 550 nm and emission wavelength of 575 nm, with red fluorescence intensity reflecting mitochondrial membrane potential levels.

### 2.21 Statistical analyses

In the realm of statistical analysis, the SPSS 16.0 software hailing from Chicago, United States, was enlisted. Data were manifested as mean ± standard deviation, sourced from a minimum of three independent replications. For comparisons between two groups, the T-test was employed, while one-way ANOVA was utilized to gauge the statistical disparities among multiple groups. A *p*-value below 0.05 signified statistical significance, thereby furnishing a solid foundation for extracting meaningful inferences from the experimental data. *In vitro* experiments were repeated in n = 3 independent experiments with triplicate wells per group. *In vivo* studies included n = 6 rats per group.

## 3 Results

### 3.1 Gypenoside XLIX alleviates the viability of neurons in the OGD model

**FIGURE 1:**
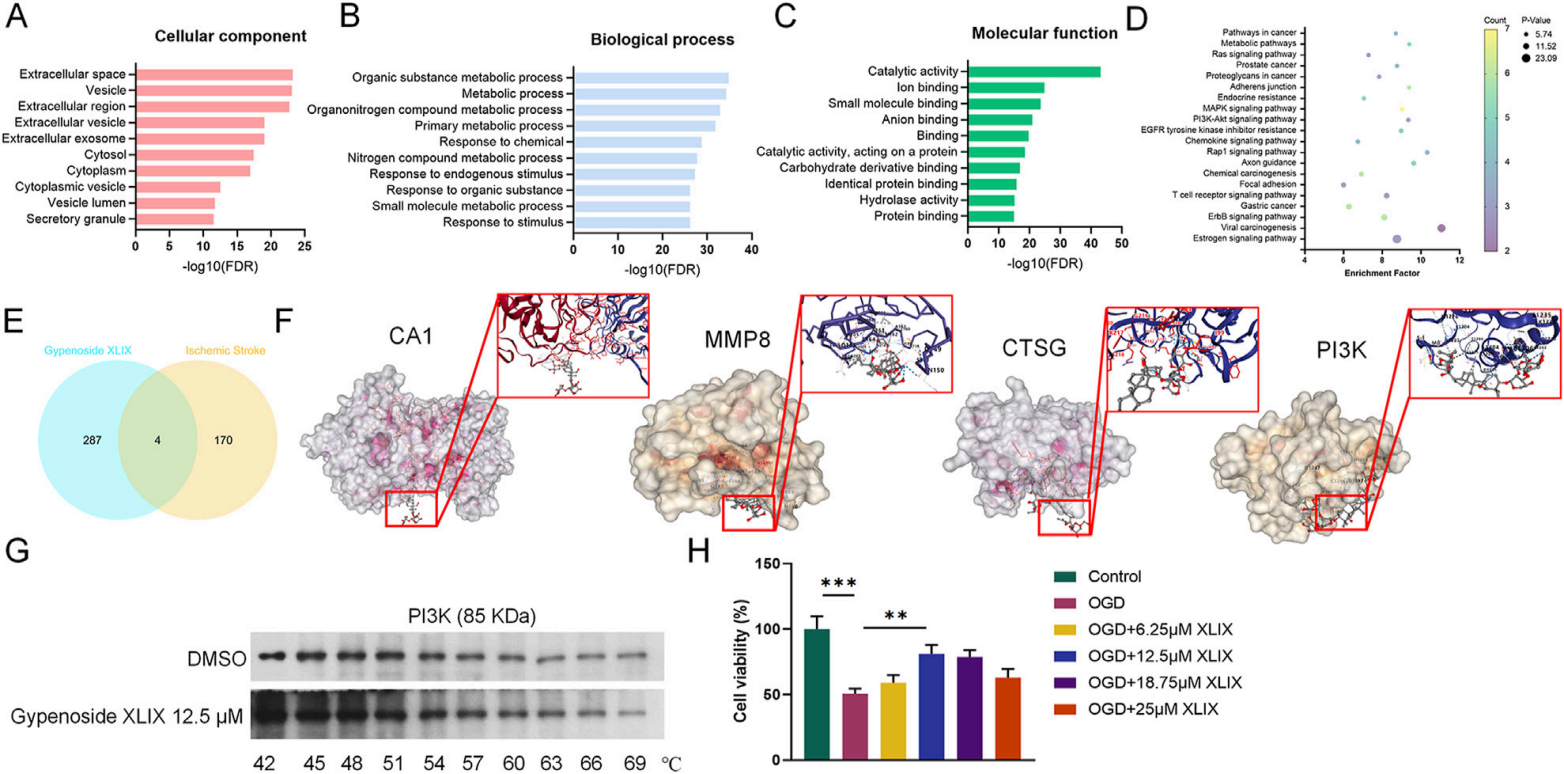
Pharmacological analysis of Gypenoside XLIX in alleviating ischemic stroke injury. **(A–C)** GO analysis of the biological process of Gypenoside XLIX. **(D)** Pathway enrichment of Gypenoside XLIX. **(E)** Venn diagram of the intersection of metabolites-disease targets. **(F)** Molecular docking results of CA1, PI3K, MMP8, and CTSG. **(G)** CETSA validation of Gypenoside XLIX-PI3K interaction. **(H)** The effect of different concentrations of Gypenoside XLIX intervention on OGD neural cell model. n = 3, **p < 0.01, ***p < 0.001.

To investigate whether Gypenoside XLIX affects stroke, online analysis software such as STRING, SymMap, and DAVID were used to predict potential targets and pathways associated with Gypenoside XLIX. Firstly, through GO analysis of biological processes, molecular functions, and cellular metabolites of Gypenoside XLIX, we found that PI3K-Akt signaling pathway were associated with Gypenoside XLIX (Figures 1A–D). Next, we found that there were 4 overlapping targets: CA1, MMP8, PI3K, and CTSG after linking the GSE22255 dataset with the predicted targets of Gypenoside XLIX (Figure 1E). At the same time, through molecular docking analysis, we found that the binding energies of hydrogen bond hydrophobic interactions between CA1, PI3K, MMP8, CTSG, and Gypenoside XLIX were −7.3 kcal/mol, −6.4 kcal/mol, −7.6 kcal/mol, −6.4 kcal/mol, and −7.1 kcal/mol respectively. The binding energy between PI3K and Gypenoside XLIX was −6.4 kcal/mol, indicating moderate binding affinity, which was comparable to other identified targets (Figure 1F). We employed the CETSA technique to investigate the interaction between Gypenoside XLIX and PI3K in depth. The experimental results demonstrated that Gypenoside XLIX treatment significantly enhanced the stability of the PI3K protein, particularly at 57°C, where this effect was most pronounced (Figure 1G). These findings reveal that Gypenoside XLIX can bind to the PI3K protein and enhance its thermal stability in neuronal cells. Finally, we constructed the OGD cell model and detected the cell viability of neuronal cells with the addition of 6.25 μM, 12.5 μM, and 18.75 μM of Gypenoside XLIX, and found that the addition of 12.5 μM Gypenoside XLIX significantly promoted (*p* < 0.01) the viability of neuronal cells in the OGD model (Figure 1H). Therefore, 12.5 μM of Gypenoside XLIX was selected for the subsequent experiments. In summary, we speculate that Gypenoside XLIX alleviates ischemic stroke by inhibiting neuronal cell apoptosis.

### 3.2 Gypenoside XLIX reduces injury to OGD neurons through PI3K

**FIGURE 2:**
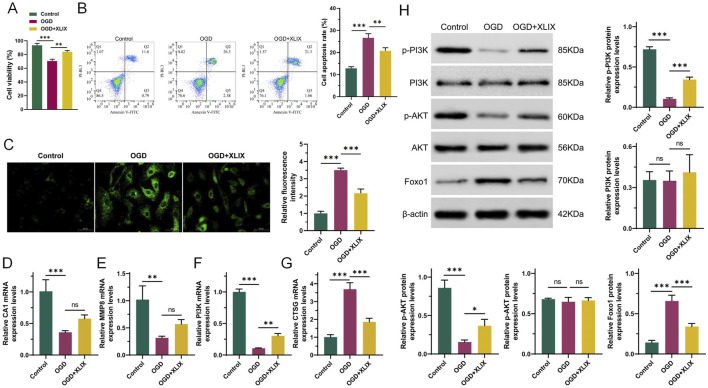
Gypenoside XLIX reduces damage to OGD neurons through PI3K. **(A)** The cell viability of neuronal cells using CCK-8. **(B)** The effect of Gypenoside XLIX intervention on the apoptosis ability of OGD neural cell model. **(C)** ROS was measured at 24 h post-reoxygenation. **(D–G)** qRT-PCR detection of expression of CA1, MMP8, PI3K, and CTSG. **(H)** Western blot detection of protein expression levels of PI3K, p-PI3K, Akt, p-Akt, and FOXO1. n = 3, *p < 0.05, **p < 0.01, ***p < 0.001.

To investigate the specific mechanism of Gypenoside XLIX in alleviating ischemic stroke, we tested the cell viability and apoptosis of neuronal cells using CCK-8 and flow cytometry assays. CCK-8 assays showed that the viability of OGD group cells was significantly lower than that of the Control group cells (*p* < 0.001). However, when treated with 12.5 μM Gypenoside XLIX, the cell viability of the OGD + XLIX group was significantly higher (*p* < 0.01) than that of the OGD group (Figure 2A). Flow cytometry experiments confirmed that the apoptosis capacity of OGD group cells was significantly higher (*p* < 0.001) than that of the Control group. When treated with 12.5 μM Gypenoside XLIX, the apoptosis capacity of the OGD + XLIX group was significantly lower (*p* < 0.01) than that of the OGD group (Figure 2B). Next, we also analyzed the changes in ROS accumulation within neuronal cells. We found that the level of ROS in the OGD + XLIX group cells treated with Gypenoside XLIX was significantly lower (*p* < 0.001) than that in the OGD group (Figure 2C). RT-qPCR testing confirmed significant low (*p* < 0.001) expression of PI3K in OGD cells treated with OGD, Gypenoside XLIX significantly reverses (*p* < 0.01) this effect (Figures 2D–G). Finally, Western blot analysis showed that the PI3K/Akt signaling pathway was activated in OGD-treated cells with Gypenoside XLIX treatment (*p* < 0.05) (Figure 2H). These results indicated that the inhibitory effects of Gypenoside XLIX on apoptosis might be related to PI3K-related pathways.

### 3.3 PI3K/AKT/FOXO1 signaling pathway promotes mitochondrial autophagy in neuronal cells

**FIGURE 3:**
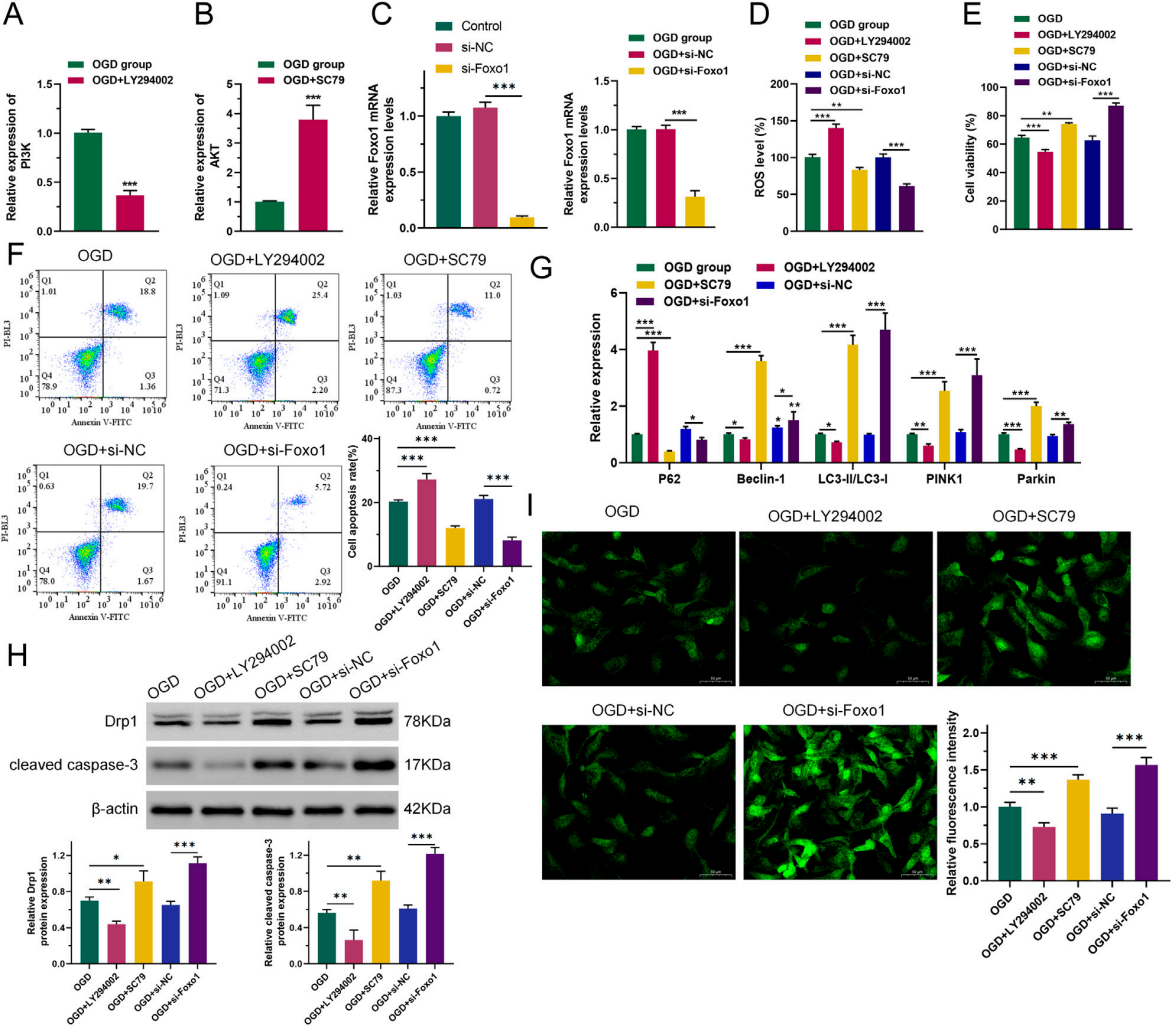
PI3K/AKT/FOXO1 signaling pathway promotes autophagy of neuronal cell mitochondria. **(A–C)** RT-qPCR detection of expression of PI3K, AKT, and FOXO1. **(D)** ROS levels were measured by DCFH-DA assay. **(E)** CCK8 was used to detect cell viability. **(F)** Flow cytometry was used to detect the ability of cells to undergo apoptosis. **(G)** qRT-PCR was used to detect the expression levels of P62, Beclin-1, LC3, PINK1, and Parkin. **(H)** Western blot was used to detect changes in protein expression of Drp1 and cleared caspase-3. **(I)** The effect of inhibition of the PI3K/AKT/FOXO1 signaling pathway on changes in intracellular total Ca^2+^ levels in OGD neurons. n = 3, *p < 0.05, **p < 0.01, ***p < 0.001.

To investigate whether the PI3K-related pathway is the main target of Gypenoside XLIX in alleviating OGD cell apoptosis, we intervened OGD cells with PI3K inhibitor LY294002 and AKT activator SC79 and constructed a FOXO1 overexpression vector for transfection of OGD cells using gene editing technology (Figures 3A–C). First, we measured the level of intracellular ROS accumulation. The levels of ROS accumulation in OGD and OGD + si-NC (negative control, non-targeting siRNA) groups were similar. However, when PI3K was inhibited, the level of intracellular ROS accumulation in OGD cells increased (*p* < 0.001). When AKT was activated and FOXO1 expression was suppressed (*p* < 0.01), the level of intracellular ROS accumulation in OGD cells decreased (Figure 3D). We also detected the viability and apoptosis of neuronal cells after interference with Gypenoside XLIX using CCK8 and flow cytometry. As shown in Figure 3E, the viability and apoptosis of cells in OGD and OGD + si-NC groups were similar. However, when PI3K was inhibited, the viability of OGD cells decreased (*p* < 0.001) and their apoptosis increased (*p* < 0.001). When AKT was activated and FOXO1 expression was suppressed, the viability of OGD cells increased (*p* < 0.01) and their apoptosis decreased (*p* < 0.001).

Next, we analyzed the expression levels of key autophagy genes. As shown in Figure 3G, the expression levels of P62, Beclin-1, LC3, PINK1, and Parkin in OGD and OGD + si-NC groups remained largely unchanged. However, when PI3K was inhibited, the expression levels of Beclin-1, LC3, PINK1, and Parkin in OGD cells significantly decreased (*p* < 0.05), while the expression of P62 significantly increased (*p* < 0.001). When AKT was activated and FOXO1 expression was suppressed, the expression levels of Beclin-1, LC3, PINK1, and Parkin in OGD cells increased (*p* < 0.05), and the expression of P62 significantly decreased (*p* < 0.05). Moreover, Western blot analysis revealed that when PI3K was inhibited, the expression of both the mitochondrial fission protein Drp1 and the apoptosis executor protein cleaved caspase-3 declined (*p* < 0.01). Conversely, when AKT was activated and FOXO1 expression was suppressed, their expression levels increased (*p* < 0.05), as depicted in Figure 3H.

Using Fluo-3 a.m. to assess intracellular total Ca^2+^ levels. As shown in Figure 3I, the Ca^2+^ level in the OGD + LY294002 group was lower (*p* < 0.01) than that in the OGD group, and the Ca^2+^ level in the OGD + SC79 group was significantly higher. The Ca^2+^ level in the OGD + si-FOXO1 group was significantly higher than that in the OGD + si-NC group. This indicated that PI3K/AKT/FOXO1 signaling increased intracellular Ca^2+^ levels in OGD cells. The obtained results imply that the PI3K/AKT/FOXO1 signaling pathway facilitates the apoptosis of neurons subjected to OGD treatment through the enhancement of mitophagy. This indicates a potential mechanism by which this signaling pathway contributes to the regulation of neuronal cell fate in the context of OGD-induced stress.

### 3.4 Gypenoside XLIX promotes mitochondrial autophagy in neuron cells through the PI3K/AKT/FOXO1 axis

**FIGURE 4:**
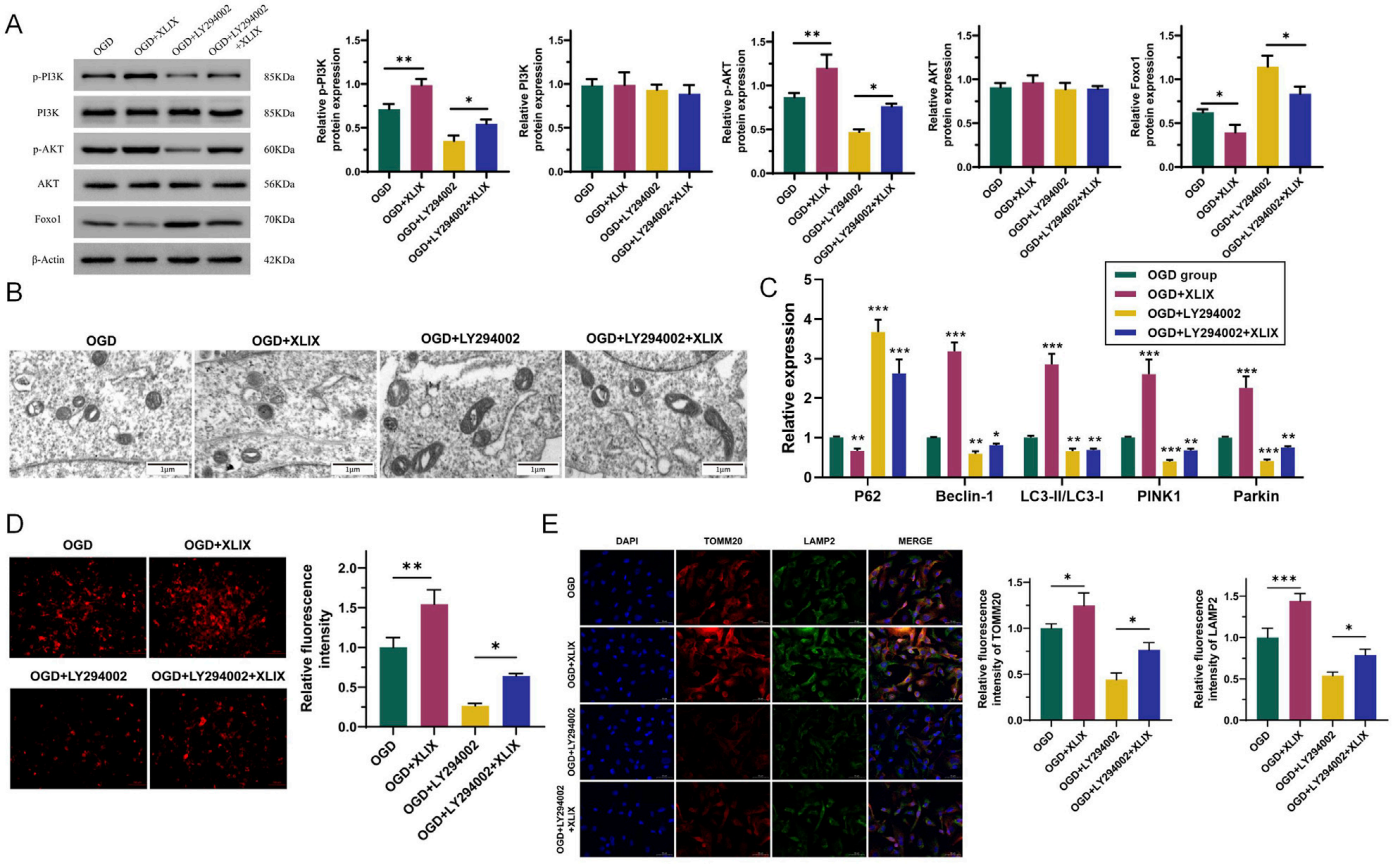
Gypenoside XLIX promotes the autophagy of neuronal mitochondria through the PI3K/AKT/FOXO1 signaling pathway. **(A)** Western blot detection of protein expression levels of PI3K, p-PI3K, Akt, p-Akt, and FOXO1. **(B)** The autophagy of mitochondria and the autophagy type of mitochondria were observed by TEM. The red arrow indicates that the mitochondria have swollen vacuoles. **(C)** The effect of Gypenoside XLIX on autophagic flux in OGD neurons. **(D)** The effect of Gypenoside XLIX on mitochondrial membrane potential in OGD neurons. **(E)** Immunofluorescence observation of co-localization of TOMM20 and LAMP2. n = 3, *p < 0.05, **p < 0.01, ***p < 0.001.

To investigate the effect of Gypenoside XLIX on the PI3K/AKT/FOXO1 signaling pathway, we detected the changes in the PI3K/AKT/FOXO1 signaling pathway in OGD cells after the addition of Gypenoside XLIX by Western blot. As shown in Figure 4A, the protein expression levels of p-PI3K and p-AKT in the OGD + XLIX group were higher (*p* < 0.01) than those in the OGD group, while the expression of FOXO1 was lower (*p* < 0.05). In the OGD + LY294002 group and OGD + LY294002+XLIX group, the protein expression levels of p-PI3K and p-AKT were lower than those in the OGD group, and the expression of FOXO1 was higher. We observed the OGD cells under different treatments by transmission electron microscopy. In Figure 4B, the mitochondria in the OGD group presented obvious deformation and swelling, with irregular shapes and ruptured cristae, and some were spherical with vacuolation (the red arrow indicates that the mitochondria have swollen vacuoles). The mitochondria in the OGD + XLIX group were rod-shaped or elliptical, with neat cristae and no expansion within the cristae. The mitochondrial deformation was most severe in the OGD + LY294002 group, with the most irregular shape. The mitochondrial state in the OGD + LY294002+XLIX group was better than that in the OGD + LY294002 group but still lower than that in the OGD + XLIX group.

Next, we further analyzed the effect of Gypenoside XLIX on the mitochondrial autophagic flux in OGD cells. In Figure 4C, the levels of Beclin-1, LC3, PINK1, and Parkin significantly increased (*p* < 0.001), and the expression of P62 significantly decreased (*p* < 0.01) in the OGD + XLIX group compared to the OGD group. However, when PI3K was inhibited, the expression levels of Beclin-1, LC3, PINK1, and Parkin in the OGD + LY294002 group and OGD + LY294002+XLIX group were significantly decreased (*p* < 0.05), and the expression of P62 significantly increased (*p* < 0.001). As shown in Figure 4D, we examined the effect of Gypenoside XLIX on mitochondrial membrane potential in OGD neurons. In the OGD + XLIX group, the fluorescence intensity was found to be significantly augmented (*p* < 0.01) compared to the OGD group. Conversely, the fluorescence intensity in the OGD + LY294002 group was significantly diminished (*p* < 0.05), and this reduction was significantly reversed because of the treatment with Gypenoside XLIX. Finally, we observed the co-localization of TOMM20 (mitochondrial marker) and LAMP2 (lysosomal marker) by immunofluorescence. Compared to the OGD group, the co-localization of TOMM20 and LAMP2 was significantly enhanced (*p* < 0.05) in the OGD + XLIX group (Figure 4E), suggesting increased mitophagosome formation. This finding was consistent with the upregulated LC3-II/LC3-I ratio and reduced p62 expression (Figure 4C), collectively indicating enhanced mitochondrial autophagic flux. TMRE staining showed that Gypenoside XLIX significantly improved mitochondrial membrane potential (MMP) in OGD cells (Figure 4D), which may be associated with autophagy-mediated clearance of damaged mitochondria.

### 3.5 Gypenoside XLIX activates PI3K/AKT/FOXO1 signaling pathway in MCAO model rats

**FIGURE 5:**
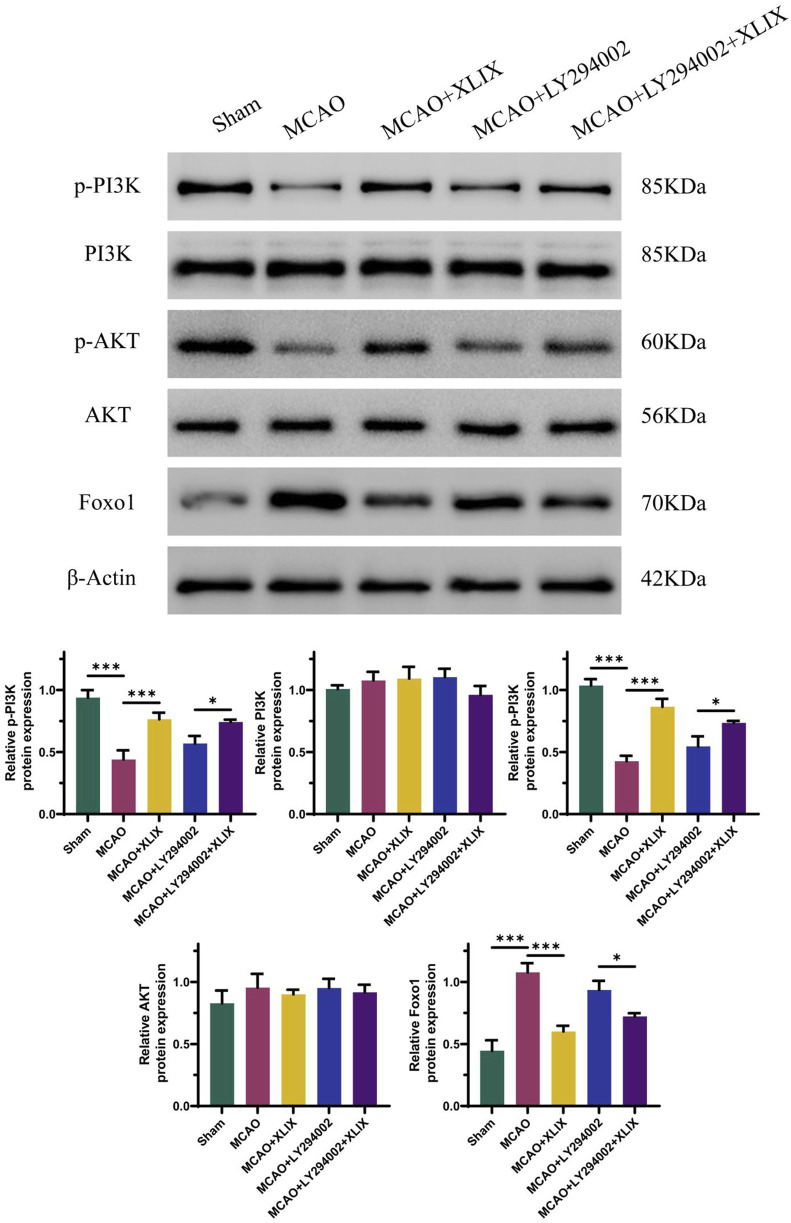
Gypenoside XLIX activates PI3K/AKT/FOXO1 signaling pathway in MCAO model rats. The detection of protein expression levels of PI3K, p-PI3K, Akt, p-Akt, and FOXO1 was carried out using Western blot technique. n = 3, *p < 0.05, ***p < 0.001.

The effect of Gypenoside XLIX on the PI3K/AKT/FOXO1 signaling pathway was examined through Western blot experiments to determine the expression levels of related proteins. In Figure 5, the levels of p-PI3K and p-AKT in the brain tissue of the MCAO + XLIX group were lower (*p* < 0.001) than those in the MCAO group, while the expression of FOXO1 was higher (*p* < 0.001) than that in the OGD group. The levels of p-PI3K and p-AKT in the brain tissue of the MCAO + LY294002 group and the MCAO + LY294002+XLIX group were less than those in the MCAO group.

### 3.6 Gypenoside XLIX mitigates ischemic stroke symptoms in MCAO model rats

**FIGURE 6:**
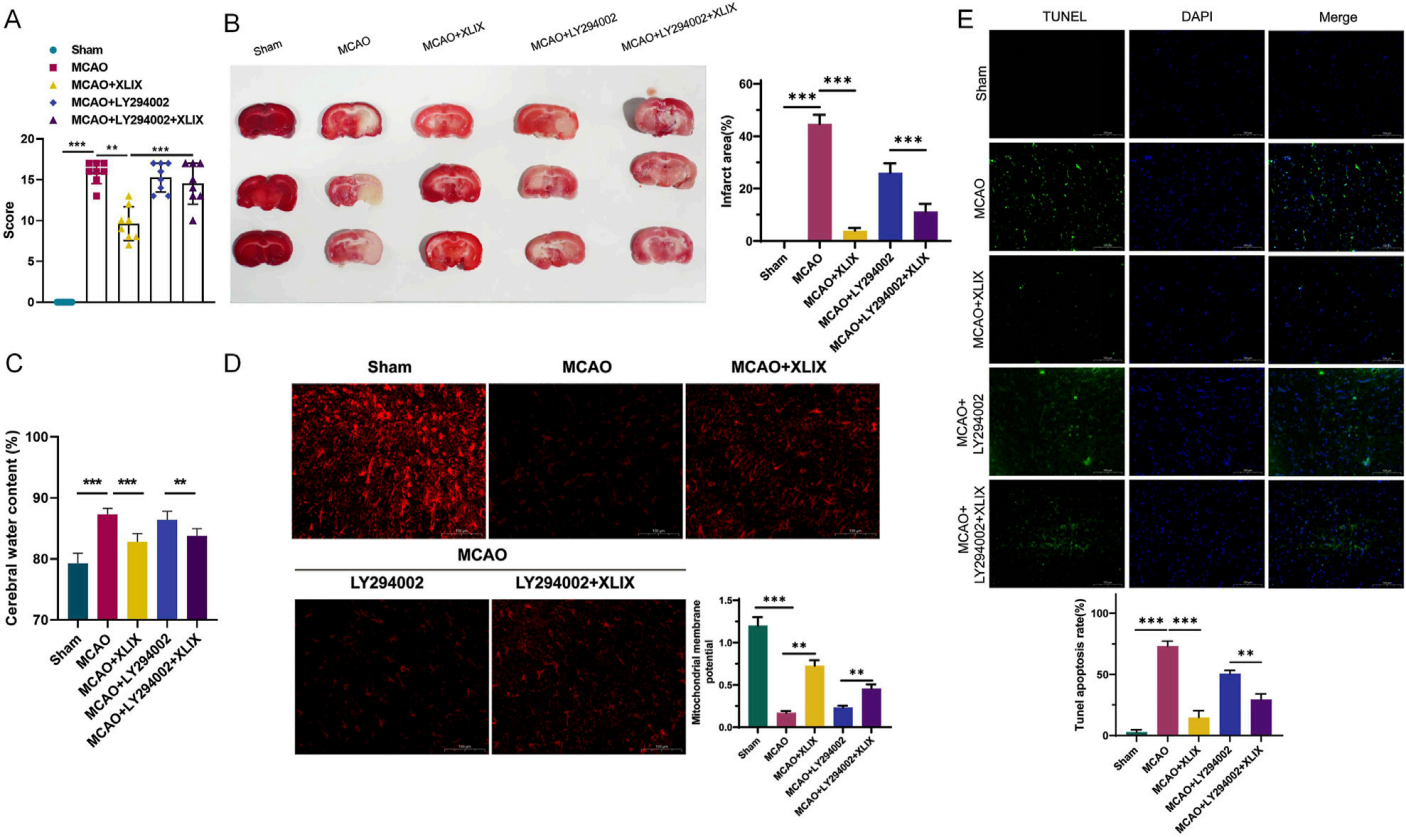
Gypenoside XLIX alleviates ischemic stroke symptoms in MCAO model rats. **(A)** The effects of cerebral ischemia-reperfusion and intervention with Gypenoside XLIX on neurological function scores in rats. **(B)** TTC staining detection of the effect of Gypenoside XLIX on the volume of cerebral infarction in rats. **(C)** Changes in brain edema rate in rats after intragastric administration of Gypenoside XLIX. **(D)** The effect of Gypenoside XLIX on mitochondrial membrane potential in the brain of rats. **(E)** TUNEL staining detection of the effect of Gypenoside XLIX on rat brain cell degeneration. n = 3, **p < 0.01, ***p < 0.001.

To analyze the neural function damage in rats after Gypenoside XLIX intervention, we assessed the changes in reflex, sensory, and motor functions of different groups of rats using a modified Neurological Score Sheet (NSS). As shown in Figure 6A, the mean NSS score of the MCAO + XLIX group was 9.68. The mean NSS scores of the MCAO + LY294002 group and the MCAO + LY294002+XLIX group were 15.25 and 14.50, respectively. As shown in Figure 6B, the infarct size in the rats after gavage administration of Gypenoside XLIX (MCAO + XLIX group) was significantly smaller (*p* < 0.001) than that in the MCAO group. Although the infarct size in the MCAO + LY294002 group and the MCAO + LY294002+XLIX group was less than that in the MCAO group, it was still higher than that in the MCAO + XLIX group. Next, we measured the changes in brain edema. The brain edema rate in the MCAO + XLIX group was significantly lower (*p* < 0.01) than that in the MCAO group. Although the brain edema rate in the MCAO + LY294002 group and the MCAO + LY294002+XLIX group was less than that in the MCAO group, it was still higher than that in the MCAO + XLIX group (Figure 6C). TMRE was used to analyze changes in the mitochondrial membrane potential of rat brain cells treated with different methods. In comparison with the Sham group, the fluorescence intensity in the MCAO group was notably decreased (*p* < 0.001). The fluorescence intensity in brain cells of the MCAO + XLIX group was significantly higher (*p* < 0.01) than that of the MCAO group (Figure 6D). The results of TUNEL staining indicated that the number of TUNEL-positive cells in the brain cells of the MCAO + XLIX group was significantly lower (*p* < 0.001) than that in the MCAO group. The quantity of TUNEL - positive cells in both the MCAO + LY294002 group and the MCAO + LY294002 + XLIX group was lower compared to that in the MCAO group (Figure 6E).

### 3.7 Effects of gypenoside XLIX intervention on oxidative stress and autophagy in rat brain through PI3K/AKT/FOXO1 signaling pathway

**FIGURE 7:**
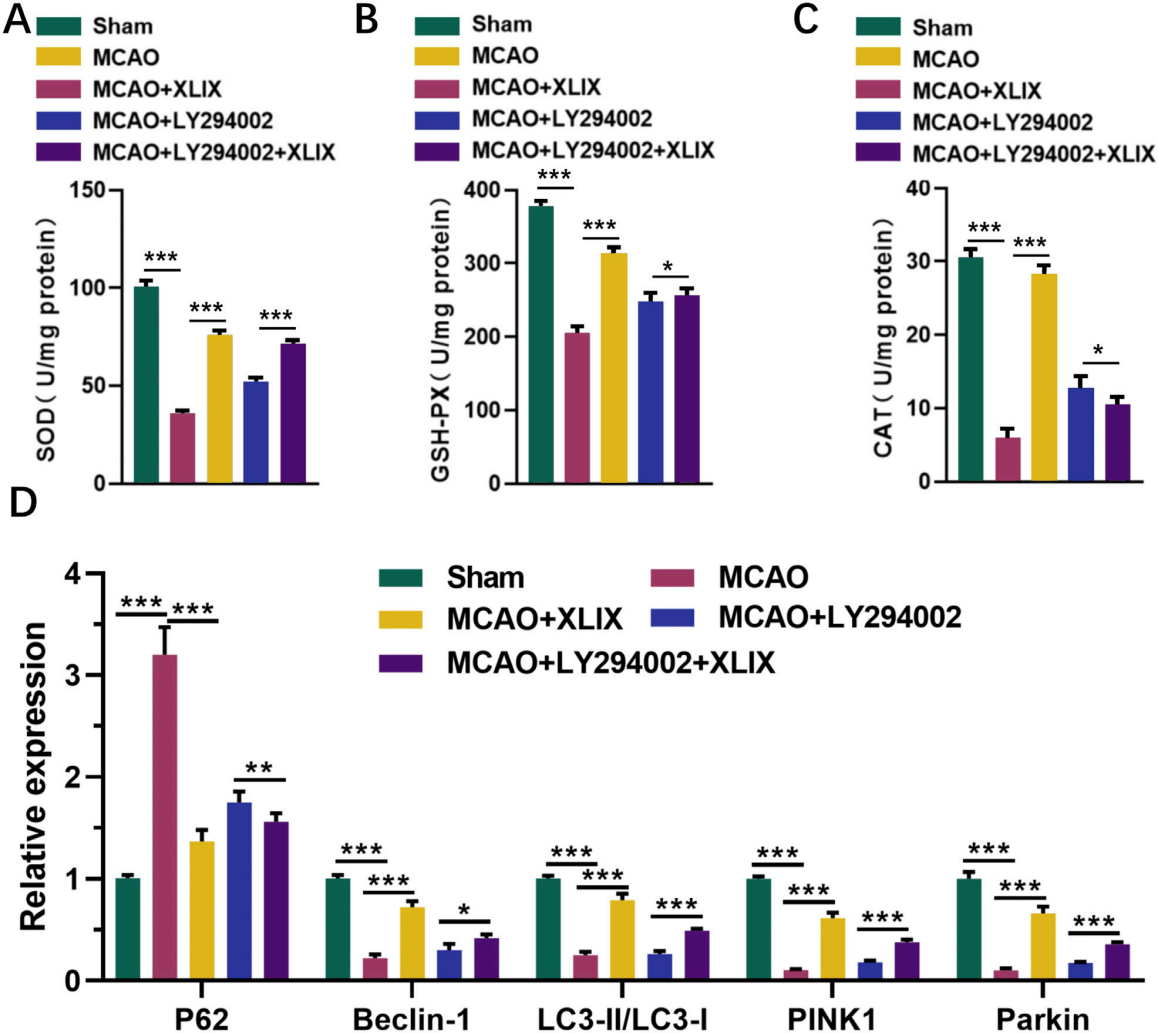
Effects of gypenoside XLIX intervention on oxidative stress and autophagy in rat brain through PI3K/AKT/FOXO1 signaling pathway. **(A–C)** The ELISA detection kit detected the expression levels of SOD, GSH-Px, and CAT in rat brain tissue. **(D)** The effect of Gypenoside XLIX on mitochondrial autophagic flux in the brain of rats. n = 3, *p < 0.05, **p < 0.01, ***p < 0.001.

Finally, we detected the levels of SOD, GSH-Px, and CAT in rat brain tissue using ELISA kits. Their levels in the brain cells of the MCAO + XLIX group were significantly higher than those in the MCAO group (*p* < 0.001). The levels of SOD, GSH-Px, and CAT in the rats of the MCAO + LY294002 group and MCAO + LY294002+XLIX group were higher (*p* < 0.05) than those in the MCAO group (Figures 7A–C). RT-qPCR analyzed the levels of key autophagy genes in the brain tissue. The expression levels of Beclin-1, LC3-II/I, PINK1 and Parkin in brain tissue of MCAO group were significantly lower (*p* < 0.001) than those of sham group, while the expression of p62 was significantly higher (*p* < 0.001) than that of sham group. The expression levels of Beclin-1, LC3-II/I, PINK1 and Parkin in brain tissue of MCAO + XLIX group were significantly higher (*p* < 0.001) than those of MCAO group, while the expression of p62 was the opposite (Figure 7D).

## 4 Discussion

Recent clinical investigations have verified that Gynostemma pentaphyllum is involved in the treatment of diverse diseases, chiefly relying on its active metabolites (Ji et al., 2018). Our research endeavors to uncover the precise mechanism through which Gynostemma pentaphyllum and its active monomer, Gypenoside XLIX, function in the treatment of ischemic stroke, as well as to explore their methods of drug application. Through bioinformatics analysis and *in vitro* experiments, we found that Gypenoside XLIX promotes mitochondrial autophagy and thereby relieves neuronal damage by enhancing the PI3K/AKT/FOXO1 signaling pathway. *In vivo* experiments have confirmed that the drug use scheme of Gypenoside XLIX significantly alleviates the brain damage caused by ischemic stroke in rats.

Modern pharmacological research shows that Gynostemma pentaphyllum contains various metabolites such as gypenosides, sterols, flavonoids, polysaccharides, amino acids, and others (Cui et al., 2021). These active substances have anti-aging, anti-fatigue, cardio-protective, hypolipidemic, liver-protective, sugar metabolism-improving, and anti-ulcer effects (Liu et al., 2023a; Guo et al., 2024; Xie et al., 2024b). Among them, total saponins of Gynostemma pentaphyllum were the main active metabolites. Gypenoside XLIX, a type of dammarane - type triterpenoid saponin, is extracted from the total saponins present in Gynostemma pentaphyllum. Liu et al. (2023b) Most of the dammarane-type triterpenoid saponins were a class of metabolites that significantly alleviate brain diseases, such as protopanaxadiol saponins, protopanaxatriol saponins, and others (Chen et al., 2023b; Wang et al., 2023). However, most existing studies still mainly focus on the protective effects of Gypenoside XLIX on the liver and kidneys (Liu et al., 2023c). Interestingly, Hao et al.'s study analyzed the plasma of crab-eating macaques after they consumed Yinaoki Granules using UPLC/ESI-Q-TOF MS and found that Gypenoside XLIX was one of the nine main metabolites that protected the PC12 cells of the macaques (Hao et al., 2020). Our study also discovered the protective effect of Gypenoside XLIX on neuron cells. Xu et al.'s study suggests that the total saponin XLIX from Gynostemma pentaphyllum alleviates sepsis-induced splenic injury mainly by inhibiting oxidative stress response to reduce the inflammatory process in rats (Xu et al., 2024). Shu and his team confirmed that oxidative stress can induce mitochondrial autophagy (Shu et al., 2021). The studies of Prakash et al. and Raffa et al. have also confirmed a correlation between stroke and mitochondrial autophagy (Prakash et al., 2021; Raffa et al., 2023). These conclusions have inspired our research. Similarly, we have investigated the changes in mitochondrial autophagy in ischemic stroke model rats after intervention with Gypenoside XLIX. In addition, we have supplemented the specific alleviating mechanism of Gypenoside XLIX on stroke. This mechanism is specifically manifested as follows: Gypenoside XLIX eliminates oxidative stress within cells, regulates mitochondrial autophagic flux, and thereby inhibits the expression of apoptosis-related proteins in rat brain tissue and neuronal cells. This process inhibits the apoptosis of neurons, protects the function of neural cells, and alleviates brain damage in MCAO rats.

PI3K phosphorylation recruits downstream signaling protein Akt. Activated Akt phosphorylates multiple substrates, thus being involved in the development of various diseases like lung cancer, heart disease, and diabetes (Chen et al., 2024; Huang et al., 2024; Zhu et al., 2024). The PI3K/Akt pathway also participates in a series of physiological activities of brain neurons. For example, UroA slows down the neurotoxicity induced by bupivacaine by inhibiting the PI3K/Akt axis (Liu and Wei, 2024). The activation of TREM2 reduces neuroinflammation and neuronal apoptosis induced by intracerebral hemorrhage in rats through the PI3K/Akt axis (Chen et al., 2020). In addition, the PI3K/Akt pathway also participates in the occurrence and progression of stroke. Xian et al.'s research has proposed that the active substances of natural drugs improving ischemic stroke through the PI3K/Akt signaling pathway has become a new trend in treatment (Liu et al., 2022). This year at the beginning, Liu et al.'s research also summarized the latest mechanisms of traditional Chinese medicine saponins affecting ischemic stroke through the PI3K/Akt signaling pathway and proposed the potential of FOXO1 as a target for the PI3K/Akt signaling pathway. Liu et al. (2023b) Foxo is a transcription factor that induces apoptosis. When AKT phosphorylates Foxo, it causes FOXO1 to move from the nucleus to the cytoplasm, thus suppressing the transcriptional function of FOXO1. Ye et al.'s research believes that the activation of the PI3K/AKT/FOXO1 signaling pathway is beneficial for peripheral nerve regeneration in patients with corpus cavernosum injury (Ye et al., 2023). Gong et al.'s research has also confirmed that the inhibition of the PI3K/AKT/FOXO1 signaling pathway leads to oxidative stress damage to dopaminergic neurons in patients with Parkinson’s disease. Our study has also verified that this pathway promotes the relief of stroke progression. Both *in vitro* cell experiments and *in vivo* adult rat experiments have confirmed our view. In addition, we have confirmed that the interference of Gypenoside XLIX is related to this process. Wu et al. found that ginsenosides from Gynostemma pentaphyllum treatment inhibit the PI3K/AKT pathway and induce apoptosis of cancer cells in gastric cancer tumors (Wu et al., 2024). Hui et al.'s study confirmed that in macrophages, ginsenosides promote the Sirt1-FOXO1 axis to mediate autophagic flux, inhibiting the uptake of oxidized LDL and the generation of foam cells (Hui et al., 2021). This study shows that Gypenoside XLIX affects the PI3K/AKT/FOXO1 signaling pathway in OGD cells and MCAO rat models. This may be achieved by regulating intracellular redox and energy metabolism. It can reduce ROS levels and improve mitochondrial membrane potential, creating a favorable environment for the activation of this signaling pathway. The research results were like some studies, all emphasizing the importance of mitochondrial autophagy and the PI3K/AKT pathway in the treatment of ischemic stroke. However, this study focuses on the impact of Gypenoside XLIX on this signaling pathway. Other studies may focus on other targets or links of this pathway.

The limitations of this study primarily lie in the use of rat models, lacking validation through human clinical trials, which leaves the feasibility and efficacy of the findings in actual clinical applications unproven. Although the study reveals the protective role of Gypenoside XLIX in regulating mitochondrial autophagy via the PI3K/AKT/FOXO1 signaling pathway, the specific molecular mechanisms require further exploration and validation. Future research should focus on clinical trials, in-depth investigation of the molecular mechanisms of Gypenoside XLIX, evaluation of its long-term effects and safety, and comparison with other stroke treatment drugs to verify its universality and reliability.

This study explores the mechanism of Gypenoside XLIX in treating stroke. The findings suggest that Gypenoside XLIX may promote mitochondrial autophagy in neuronal cells and reduce intracellular ROS accumulation by regulating the PI3K/AKT/FOXO1 signaling pathway, which could potentially alleviate stroke-related damage. These results indicate the therapeutic potential of Gypenoside XLIX for stroke and provide new insights for clinical treatment strategies.

## Data Availability

The raw data supporting the conclusions of this article will be made available by the authors, without undue reservation.
