# Supplementary material for: Gypenoside XLIX inhibiting PI3K/AKT/FOXO1 signaling pathway mediated neuronal mitochondrial autophagy to improve patients with ischemic stroke
Source: Front Pharmacol. 2025 Aug 21;16:1600435. doi: 10.3389/fphar.2025.1600435 (PMC12408567; doi:10.3389/fphar.2025.1600435)
Supplement: Supplementary file 1 [file Table1.docx]

**Supplementary Table 1** Modified NSS Score Scale

| Neurological defects | | | score |
| --- | --- | --- | --- |
| Exercise test | | | |
|  | Tail-flick test | | |
|  |  | Forelimb flexion | 1 |
|  |  | Hindlimb flexion | 1 |
|  |  | The head deviates from the vertical axis within 30 seconds>10° | 1 |
|  | Place the rat on the floor (normal=0; maximum=3) | | |
|  |  | Normal walking | 0 |
|  |  | Unable to walk in a straight line | 1 |
|  |  | Rotate towards the paraplegic side | 2 |
|  |  | Tilting towards the paraplegic side | 3 |
| Sensory test | | | |
|  | Placement test (visual and tactile test) (positive for delayed reaction when tilted at a 45 ° angle 10cm away from the desktop and close to the desktop) | | 1 |
|  | Proprioceptive test (deep sensation, pressing mouse claws towards the edge of the table to stimulate limb muscles) | | 1 |
|  | Balance beam test (normal value=0; maximum value=6) | | |
|  |  | Stable balance posture | 0 |
|  |  | Grasp the edge of the balance beam tightly | 1 |
|  |  | Holding onto the balance beam tightly, one limb hangs down from the balance beam | 2 |
|  |  | Hold onto the balance beam tightly, with both limbs falling or rotating from the balance beam (>60 s) | 3 |
|  |  | Attempted to balance on the balance beam but fell (>40 s) | 4 |
|  |  | Attempted to balance on the balance beam but fell (>20 s) | 5 |
|  |  | Falling; Not attempting to balance on the balance beam (<20 s) | 6 |
| Loss of reflexes and abnormal movements | | | |
|  | Auricular reflex (shaking the head when in contact with the external ear canal) | | 1 |
|  | Corneal reflex (blinking when lightly touched with cotton thread) | | 1 |
|  | Panic reflex (has a motor response to the noise of fast-moving cardboard) | | 1 |
|  | Epilepsy, muscle spasms, muscle tone disorders | | 1 |

**Supplementary Table 2** Primer Sequences for the Target Gene

| Name | Sequence (5’-3’) |
| --- | --- |
| P62 forward | GCTGGTGCATCCATCAGTACC |
| P62 reverse | GCTGAAGGGTTGTTACCTCTGT |
| Beclin-1 forward | GCCCAGACAGGACTCTCTTAG |
| Beclin-1 reverse | TGAACACACTTGCCAGTCTTC |
| LC3 forward | CCCAAGCGTCAGACCCTTC |
| LC3 reverse | GGGGAACTTTGCCCGGATT |
| PINK1 forward | GGCCTAAGCCCCGATGTAAAA |
| PINK1 reverse | GGCCATTTGAGGCACAAACA |
| Parkin forward | TCCTGGGTGTTCGACAGCTA |
| Parkin reverse | CCGAATCCTTCACGAAGTCCT |
| PI3K forward | TCTGGAGAAGATCCTGGAG |
| PI3K reverse | GAGCACAGCAAATAGAGCA |
| AKT forward | TCACCCAGTGACAACTCAG |
| AKT reverse | AAACTCGTTCATGGTCACAC |
| FOXO1 forward | CCGCGTCTCCTGGTACTCT |
| FOXO1 reverse | GCAGGCTCAGGTTGCTCATA |
| GAPDH forward | AACTTTGGCATTGTGGAAGGGCTC |
| GAPDH reverse | TGGAAGAGTGGGAGTTGCTGTTGA |
